# Reduced osteoblast activity in the mice lacking TR4 nuclear receptor leads to osteoporosis

**DOI:** 10.1186/1477-7827-10-43

**Published:** 2012-06-07

**Authors:** Shin-Jen Lin, Hsin-Chiu Ho, Yi-Fen Lee, Ning-Chun Liu, Su Liu, Gonghui Li, Chih-Rong Shyr, Chawnshang Chang

**Affiliations:** 1 George Whipple Lab for Cancer Research, Departments of Pathology, Urology, Radiation Oncology, and The Wilmot Cancer center, University of Rochester Medical Center, Rochester, NY, 14642, USA; 2 Sex Hormone Research Center, China Medical University/Hospital, Taichung, 404, Taiwan

**Keywords:** TR4, Nuclear receptor, Bone, Osteoporosis

## Abstract

**Background:**

Early studies suggested that TR4 nuclear receptor might play important roles in the skeletal development, yet its detailed mechanism remains unclear.

**Methods:**

We generated TR4 knockout mice and compared skeletal development with their wild type littermates. Primary bone marrow cells were cultured and we assayed bone differentiation by alkaline phosphatase and alizarin red staining. Primary calvaria were cultured and osteoblastic marker genes were detected by quantitative PCR. Luciferase reporter assays, chromatin immunoprecipitation (ChIP) assays, and electrophoretic mobility shift assays (EMSA) were performed to demonstrate TR4 can directly regulate bone differentiation marker osteocalcin.

**Results:**

We first found mice lacking TR4 might develop osteoporosis. We then found that osteoblast progenitor cells isolated from bone marrow of TR4 knockout mice displayed reduced osteoblast differentiation capacity and calcification. Osteoblast primary cultures from TR4 knockout mice calvaria also showed higher proliferation rates indicating lower osteoblast differentiation ability in mice after loss of TR4. Mechanism dissection found the expression of osteoblast markers genes, such as ALP, type I collagen alpha 1, osteocalcin, PTH, and PTHR was dramatically reduced in osteoblasts from TR4 knockout mice as compared to those from TR4 wild type mice. *In vitro* cell line studies with luciferase reporter assay, ChIP assay, and EMSA further demonstrated TR4 could bind directly to the promoter region of osteocalcin gene and induce its gene expression at the transcriptional level in a dose dependent manner.

**Conclusions:**

Together, these results demonstrate TR4 may function as a novel transcriptional factor to play pathophysiological roles in maintaining normal osteoblast activity during the bone development and remodeling, and disruption of TR4 function may result in multiple skeletal abnormalities.

## Background

The TR4 nuclear receptor belongs to the nuclear receptor superfamily, which is comprised of transcription factors that are related by sequence and structure [1]. As transcription factors, nuclear receptors control the expression of target genes and thereby direct developmental, physiological, and behavioral responses from the cellular level to that of the whole organism. TR4 is closely related to the TR2, retinoid X receptor, COUP-TF, and HNF4 in sequence and structure [2], and all of them bind to AGGTCA DNA sequence motifs in direct repeat (DR) orientation, with variable spacing, in the promoters of their target genes [1,3-6]. Via knocking out TR4 (TR4^−/−^), a complex set of phenotypic abnormalities were found to exist in the TR4^−/−^ mouse, including significant growth retardation [7], defects in female reproductive function [8] and maternal behavior [7], impaired cerebella function [9,10], reduced sperm production [11], and reduced myelination [12], as well as abnormalities in glucose [13], lipid metabolism [14], and foam cell formation [15].

In this study, we reported that TR4 is involved in regulation of osteoblast activity via regulating osteocalcin expression. TR4 might represent a novel transcriptional factor involved in the bone remodeling network and provide a potential linkage to the pathogenesis of osteoporosis in human disease.

## Methods

### Mouse studies

All animal procedures were approved by the Animal Care and Use Committee of the University of Rochester. TR4^−/−^ mice used in this study were generated from heterozygous breeding pairs provided by Lexicon Genetics and genotyped as previously described [16].

### Cell culture

Bone marrow cells were isolated from 10 weeks old TR4 wild type (TR4^+/+^) and TR4^−/−^ mice. Cells were cultured in 2 mL of a modified essential medium (α-MEM) containing 10% fetal bovine serum (FBS) at 5x10^6^ cells/well in 6-well plates. After 7 days in culture, the media was replaced with media containing 10 mM β-glycerophosphate and 50 mg/mL ascorbic acid. This media was changed every 2 days thereafter. On day 21 after plating, cells were fixed for alkaline phosphatase (ALP) and alizarin red staining or harvested for mRNA isolation using the RNeasy Mini Kit (Qiagen).

Mouse calvaria cells were obtained from neonatal mice 2–4 days after birth using sequential collagenease digestion. Briefly, calvaria were digested with trypsin/EDTA, then 1 mg/mL collagenase. Cells were collected by centrifugation, and cultured in proliferation media (DMEM with 20% FBS). Calvaria cells were cultured until 80% confluence and proliferation media was replaced by differentiation media (α-MEM containing 10% FCS, 2 mmol/L glutamine, 50 mg/mL ascorbic acid, and 10 mmol/L b-glycerol phosphate).

### Real-time PCR

Total RNA was isolated from TR4^−/−^ and TR4^+/+^ mice calvaria cultures using TRIzol Reagent (Invitrogen). The relative abundance of target mRNA was quantified relative to the control β-actin gene expression from the same reaction. Real-time PCR quantification amplifications of reverse-transcribed first strand DNA samples were performed using the iCycler iQ PCR cycler (Bio-Rad). Relative quantification of PCR products were based on value differences between the target and β-actin control using the 2^ΔΔCT^ method.

### The electrophoretic mobility shift assay (EMSA) & chromatin immunoprecipitation (ChIP) assay

TR4 protein for EMSA was transcribed and translated in TNT reticulocyte lysates (Promega). For the antibody super shift assay, the mixtures were incubated for 15 min in the presence or absence of a mouse anti-TR4 monoclonal antibody. The protein/DNA complexes were analyzed on a 5% native polyacrylamide gel. The results were visualized by autoradiography (Storm PhosphorImaging System, Amersham Pharmacia).

ChIP assays were performed in MC3T3 cells. Immunoprecipitations were performed at 4°C overnight, with 2 μg monoclonal antibody.

### Bone tissue preparation

The femurs were collected and excess muscle and soft tissues were excised. The bone specimens were fixed in 10% neutral buffered formalin. The specimens were decalcified for 21 days in 14% EDTA (pH 7.2), embedded in paraffin, and sectioned at thickness of 3 μm. The sections were stained as described previously [17].

## Results

### Mice lacking TR4 develop skeletal abnormalities

**Figure 1:**
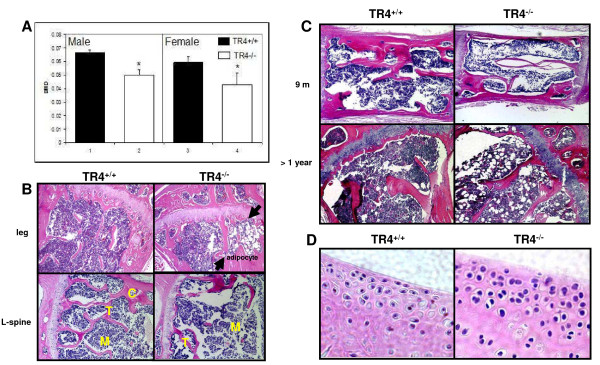
**Skeletal abnormalities in TR4**^**−/−**^**mice.*A***: Reduced bone mineral density in TR4^−/−^ mice, in both genders. DEXA scan of 6 pairs and 5 pairs of 6 months old TR4^−/−^ male and female mice, respectively. The average bone density was compared. ***B***: Bone histology. Reduced bone volume in TR4^−/−^ long bone (upper panel) and spinal column (L-spine, lower panel). HE staining is from 6 month old TR4^+/+^ and TR4^−/−^ mice legs and lumbar-spines. M: bone marrow; T: trabeculae; C: cortex. Accumulation of adipocytes found in TR4^−/−^ mice. Arrows indicate that there is more fat accumulation in TR4^−/−^ mice bone marrow. ***C***: Continuing reduced bone volume at 9 months and over one year old TR4^−/−^ mice. ***D***: Osteoarthritis in TR4^−/−^ mice. 6 months old TR4^−/−^ knee joint shows signs of osteoarthritis.

Knockout of TR4 in mice results in growth retardation with reduction of circulating growth hormone [16]. One of the possible mechanisms is through regulation of skeletal growth. However, skeletal abnormalities can result from a direct effect on bone formation or remodeling. Clinically, the decreased bone mass observed in age-related osteoporosis is often accompanied by increased marrow adipose tissue. We found mice lacking TR4 might develop osteoporosis. As shown in Figure 1A, DEXA scan of TR4^+/+^ and TR4^−/−^ mice at 6 months of age revealed a significant reduction in bone mineral density in both male and female mice. Bone histology of TR4^−/−^ trabecular bone showed significant increased marrow adipocyte deposits (Figure 1B, upper panel, arrows). In addition, reduced bone volume was found in the spinal column (L-spine) of the TR4^−/−^ mice as compared to TR4^+/+^ mice (Figure 1B, lower panel). Reduced trabecular bone volume and increased adipocyte deposits were also found in 9 months old and over a year old TR4^−/−^ mice (Figure 1C). Interestingly, a few TR4^−/−^ mice at age of 6 months also started to develop signs of osteoarthritis with increasing superficial zone cells and disruption of articular surface. (Figure 1D). Together, results from Figure 1A-D suggest loss of TR4 may lead to the development of skeletal abnormalities.

### Reduced osteoblast differentiation in TR4^−/−^ mice

**Figure 2:**
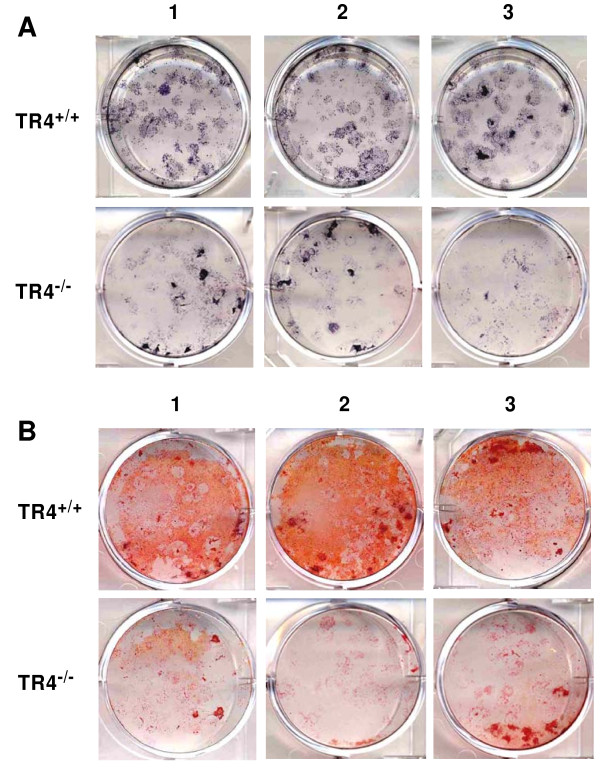
**TR4**^**−/−**^**bone marrow cultures have reduced osteoblast differentiation.** Bone marrow cells were isolated from 10 weeks old TR4^−/−^ and control TR4^+/+^ mice. Cells were cultured in 2 mL α-MEM containing 10% FBS at 5x10^6^ cells/well in 6-well plates. After 7 days, the media was replaced with media containing β-glycerophosphate and ascorbic acid to induce osteoblast differentiation. Colonies were stained on day 21 after plating by alkaline phosphatase (***A***) and alizarin red ( ***B***). The assays were performed in triplicate.

All above observed skeletal abnormal phenotypes suggest that the TR4^−/−^ mice may develop accelerated bone loss, which is similar to human osteoporosis. To determine whether TR4 has a direct impact on the osteoblastic activity, bone marrow progenitor cells extracted from TR4^−/−^ and TR4^+/+^ mice were isolated and placed in culture for colony formation assays. After 7 days, osteogenic media containing β-glycerol-phosphate and ascorbic acid was added to the cultures. ALP and alizarin red staining was performed, and ALP staining showed fewer colonies formed with reduced ALP density present in the TR4^−/−^ bone marrow cells as compared to TR4^+/+^ littermates (Figure 2A). Similarly, alizarin red staining was decreased in the TR4^−/−^ bone marrow cells compared with TR4^+/+^ control (Figure 2B). Together, results from Figure 2A-B suggests TR4 can regulate bone differentiation.

### Reduction of osteoblastic differentiation and associated genes in calvaria from TR4^−/−^ mice

**Figure 3:**
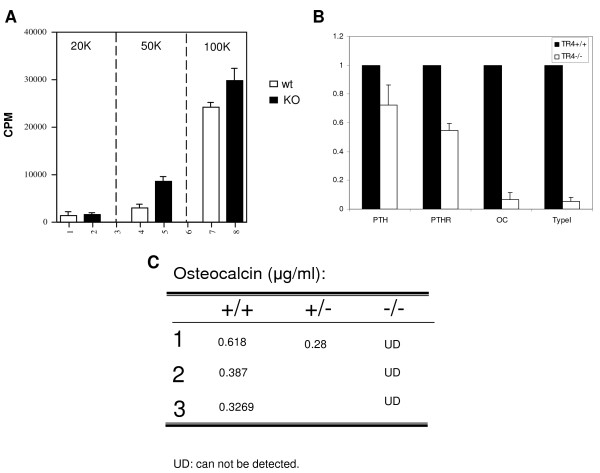
**Characterization of primary calvaria cultures from TR4**^**−/−**^**vs. TR4**^**+/+**^**mice.*A***: Proliferation rate was compared between primary calvaria cultures of TR4^−/−^ and TR4^+/+^ pups by ^3^ H-thymidine incorporation assay. The 20 K, 50 K, and 100 K numbers represent different cell number seeding density. ***B***: Quantification of osteoblastic marker gene expression. Total RNA were harvested from the primary calvaria cells of TR4^−/−^ and TR4^+/+^ mice, and converted to first strand cDNA, and subjected to q-PCR. The relative expression fold of the genes from TR4^−/−^ mice, as compared to TR4^+/+^ mice, were calculated. ***C***: The serum levels of osteocalcin from TR4^−/−^ and TR4^+/+^ mice were determined by ELISA.

To further confirm TR4 roles in osteoblast differentiation, primary osteoblasts from 2–4 day old TR4^−/−^ mice *vs.* TR4^+/+^ mice calvaria were isolated and cultured. Cell proliferation rates were compared with different seeding densities (from 2x10^4^-1x10^5^ cells), and results showed that calvaria cells from TR4^−/−^ mice had higher proliferation rates than those from TR4^+/+^ mice, indicating lower osteoblast differentiation ability in TR4^−/−^ mice (Figure 3A).

Quantitative real-time PCR analysis was then performed to determine the expression of genes associated with osteoblast activity. As shown in Figure 3B, all the expression levels of osteoblast marker genes, including PTH, PTHR, osteocalcin, and type I collagen alpha 1 were found to be dramatically reduced in osteoblasts from TR4^−/−^ mice as compared to those from TR4^+/+^ mice. This reduction of osteoblastic gene expression also reflected undetectable serum concentrations of osteocalcin, a bio-marker for osteoblast activity, found in TR4^−/−^ mice (Figure 3C), suggesting TR4 may be able to regulate bone differentiation through those differentiation markers.

### TR4 modulates osteocalcin gene at transcriptional level

**Figure 4:**
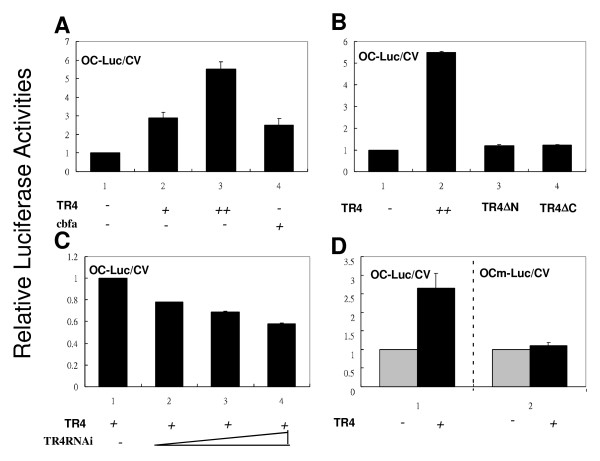
**TR4 transcriptionally regulates osteocalcin expression.*A***: Overexpression of TR4 promotes OC-Luc activity. Increasing amounts of pCMX-TR4 (1 and 2 μg, marked as + and ++, respectively) and the positive control pCMX-cbfa were transfected into CV-1 cells, followed by luciferase reporter assay (OC-Luc/CV). ***B***: Overexpressing TR4 mutants (TR4▽N or TR4▽C) diminishes osteocalcin promoter activity. ***C***: Knockdown of TR4 by siTR4 reduces osteocalcin promoter activity. Retro-siTR4 was infected into OC-Luc transfected CV-1 cells, followed by a reporter gene assay. ***D***: Mutation of osteocalcin promoter diminishes promoter activity. A mutation of TR4RE in osteocalcin (OCm-Luc/CV) was co-transfected with TR4, and followed by a reporter gene assay. All the experiments were repeated three times independently.

To further dissect the molecular mechanism by which TR4 can modulate those osteoblast marker genes, we focused on the osteocalcin gene regulation, since osteocalcin is secreted only by osteoblasts and thought to play an important role in the regulation of osteoblastic activity in bone mineralization and calcium ion homeostasis [18,19]. As a transcriptional factor, TR4 is known to bind to AGGTCA-like DR motif located in the targeted gene’s promoter region to regulate its expression. It has been reported that the 5′-promoter region of the osteocalcin gene contains the DR3 motif that can be bound and regulated by vitamin D receptor (VDR). This DR3 motif is highly conserved among species and the sequences are very similar among rat, mouse, and human promoters [20,21]. Since TR4 and VDR share the same response element [5], it is possible that TR4 can also bind and regulate osteocalcin at the transcriptional level. As shown in Figure 4A, TR4 induced osteocalcin promoter luciferase (OC-Luc) activity in a dose-dependent manner. Cbfa, a known osteocalcin transcriptional factor [22], was used as a positive control.

Furthermore, we found deletion of the N- and C-terminals of TR4 (TR4▽N and TR4▽C, respectively) resulted in loss of the TR4 ability to transactivate OC-Luc (Figure 4B), suggesting the full-length of TR4 is required for this regulation. This conclusion was further confirmed showing TR4-mediated osteocalcin promoter transcriptional activation was reduced in the TR4-siRNA knockdown cells in a dose dependent manner (Figure 4C). Importantly, as the third G within the DR motif is important for TR4 binding, we therefore mutated this position from GGGTGAatgAGGACA to GGATGAatgAGTACA and as expected, we found mutated TR4RE in OC-Luc (OCm-Luc) was no longer able to transactivate luciferase activity (Figure 4D).

Together, results from Figure 4A-D suggested that TR4 modulated bone formation via regulating osteocalcin gene expression at the transcriptional level.

### TR4 binds to the regulatory sequence of osteocalcin gene *in vitro* and *in vivo*

**Figure 5:**
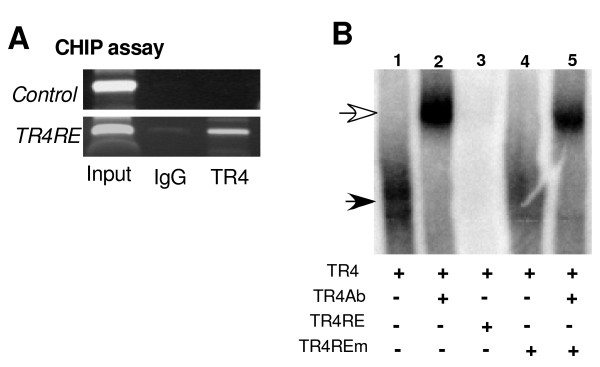
**TR4 binds to TR4RE in osteocalcin promoter.*A***: The binding of TR4 to TR4RE (DR3) in osteocalcin was demonstrated by ChIP assay. Anti-TR4 antibody was used to pull-down the DNA-protein complex, and binding sequence was amplified by the specific primers that cover the TR4RE. ***B***: EMSA. TR4 proteins were translated by TNT rabbit reticular lysate and applied to the gel shift assay. The probe (TR4RE) was labeled with ^32^P, and TR4 antibody was used for the super shift (lane 2). 100 x cold competition with wild type TR4RE (lane 3), or TR4REm (lane 4, 5) were applied.

To further prove TR4 can bind directly to this DR3 DNA fragment, the TR4 response element (TR4RE) that is located at −465 to −437 of the osteocalcin promoter, we performed ChIP assay. As shown in Figure 5A, TR4 antibody, but not IgG control, could pull-down the DNA-protein complex containing the DR3 that could be amplified by the specific primers covering that region.

To further test the TR4-TR4RE interaction *in vitro*, EMSA was applied. As shown in Figure 5B, TR4 can form a complex with TR4RE, and this TR4-TR4RE can be super shifted by adding TR4 antibody (lane 2) [4]. The specific TR4-DNA-protein complex was further competed out by 100 X unlabeled TR4RE (lane 3), but not mutant TR4RE (TR4REm) (lane 4 and 5), suggesting TR4 can physically bind to osteocalcin promoter region to transcribe its expression.

## Discussion

We found that TR4 directly influences osteoblast activity with reduced osteoblast differentiation found in bone marrow progenitor cells isolated from TR4^−/−^ mice as compared to TR4^+/+^ mice. This reduction of bone marrow progenitor cells differentiation could then contribute to osteoporosis developed in their later life. Our data are in agreement with the critical role of bone marrow progenitor cells in the senile osteoporosis where depletion and/or inhibition of bone marrow progenitor cells differentiation and activation could cause osteoporosis. TR4 represents a novel factor that controls differentiation of bone marrow progenitor cells allowing for osteoblast formation. And the depletion of TR4 in mice results in premature aging accompanied with osteoporosis in both genders. TR4^−/−^ mice display a significant adipocyte accumulation in their trabecular bones, indicating that blocking TR4 function can also alter the balance between adipogenesis and osteogenesis, and that bone formation is inhibited. These findings make TR4 a candidate gene to monitor bone activity.

TR4 transcript levels did not alter when undifferentiated embryonic stem cells were induced into adipocytes and osteoblasts [23], suggesting that the environmental/exogenous factors that control TR4 activity might be critical for controlling the bone marrow progenitor cells activity to affect osteoblast differentiation. Therefore, identifying its upstream modulator(s) offers novel therapeutic and/or preventive approaches to target senile osteoporosis. However, in contrast to TR4, it is reported that attenuation of PPAR gamma function could promote osteogenesis, such that bone formation can be enhanced [24]. Since both TR4 and PPAR gamma are members of the nuclear receptor superfamily and share the similar regulatory DNA response elements and some upstream modulator(s), it will be of interest to dissect the activity/specificity of both receptors in the process of early bone marrow progenitor cells differentiation that might have a great impact on the osteoblast activity.

## Conclusions

Together, these results demonstrate TR4 may function as a novel transcriptional factor to play its pathophysiological roles in maintaining normal osteoblast activity during the bone development and remodeling, and disruption of TR4 function may result in multiple skeletal abnormalities.

## Competing interests

The authors declare that they have no competing interests.

## Authors’ contributions

ShL participated in the *in vivo* and *in vitro* assays and drafted the manuscript. HH carried out the *in vivo* and *in vitro* assays. YL participated in experimental design and drafted the manuscript. NL carried out the *in vitro* assays. SuL participated in experimental design. GL and CS participated in experimental design and discussion. CC conceived of the study, and participated in its design and coordination and helped to draft the manuscript. All authors read and approved the final manuscript.

## References

[B1] Lee YF Lee HJ Chang C Recent advances in the TR2 and TR4 orphan receptors of the nuclear receptor superfamily J Steroid Biochem Mol Biol 2002 81 4–5 291 308 12361719 10.1016/s0960-0760(02)00118-8

[B2] Chang C Da Silva SL Ideta R Lee YF Yeh S Burbach JPH Human and rat TR4 orphan receptors specify a subclass of the steroid receptor superfamily Proceedings of the National Academy of Sciences USA 1994 1994 6040 6044 10.1073/pnas.91.13.6040 PMC44133 8016112

[B3] Lee YF Pan HJ Burbach PH Morkin E Chang C Identification of direct repeat 4 as a positive regulatory element for the human TR4 orphan receptor: a modulator for the thyroid hormone target genes J Biol Chem 1997 272 12215 12220 10.1074/jbc.272.18.12215 9115296

[B4] Lee YF Young WJ Burbach JPH Chang C Negative feedback control of the retinoid-retinoic acid/retinoid X receptor pathway by the human TR4 orphan receptor, a member of the steroid receptor superfamily J Biol Chem 1998 273 13437 13443 10.1074/jbc.273.22.13437 9593676

[B5] Lee YF Young WJ Lin WJ Shyr CR Chang C Differential regulation of direct repeat 3 vitamin D3 and direct repeat 4 thyroid hormone signaling pathways by the human TR4 orphan receptor J Biol Chem 1999 274 16198 16205 10.1074/jbc.274.23.16198 10347174

[B6] Lee Y-F Shyr CR Thin TH Lin WJ Chang C Convergence of two repressors through heterodimer formation of androgen receptor and testicular orphan receptor-4: a unique signaling pathway in the steroid receptor superfamily Proceedings of the National Academy of Sciences USA 1999 96 14724 14729 10.1073/pnas.96.26.14724 PMC24715 10611280

[B7] Collins LL Lee YF Heinlein CA Liu NC Chen YT Shyr CR Meshul CK Uno H Platt KA Chang C Growth retardation and abnormal maternal behavior in mice lacking testicular orphan nuclear receptor 4 Proc Natl Acad Sci USA 2004 101 42 15058 15063 10.1073/pnas.0405700101 15477591 PMC524065

[B8] Chen LM Wang RS Lee YF Liu NC Chang YJ Wu CC Xie S Hung YC Chang C Subfertility with defective folliculogenesis in female mice lacking testicular orphan nuclear receptor 4 Mol Endocrinol 2008 22 4 858 867 10.1210/me.2007-0181 18174360 PMC2725750

[B9] Chen YT Collins LL Uno H Chang C Deficits in motor coordination with aberrant cerebellar development in mice lacking testicular orphan nuclear receptor 4 Mol Cell Biol 2005 25 7 2722 2732 10.1128/MCB.25.7.2722-2732.2005 15767677 PMC1061629

[B10] Chen YT Collins LL Chang SS Chang C The roles of testicular orphan nuclear receptor 4 (TR4) in cerebellar development Cerebellum 2008 7 1 9 17 10.1007/s12311-008-0006-3 18418664

[B11] Mu X Lee Y Liu N Chen Y Kim E Shyr C Chang C Targeted inactivation of Testicular nuclear Orphan Receptor 4 delays and disrupts late meiotic prophase and subsequent meiotic divisions of spermatogenesis Mol Cell Biochem 2004 24 5887 5899 10.1128/MCB.24.13.5887-5899.2004 PMC480911 15199144

[B12] Zhang Y Chen YT Xie S Wang L Lee YF Chang SS Chang C Loss of testicular orphan receptor 4 impairs normal myelination in mouse forebrain Mol Endocrinol 2007 21 4 908 920 17227886 10.1210/me.2006-0219

[B13] Liu NC Lin WJ Kim E Collins LL Lin HY Yu IC Sparks JD Chen LM Lee YF Chang C Loss of TR4 orphan nuclear receptor reduces phosphoenolpyruvate carboxykinase-mediated gluconeogenesis Diabetes 2007 56 12 2901 2909 10.2337/db07-0359 17827404

[B14] Kim E Liu NC Yu IC Lin HY Lee YF Sparks JD Chen LM Chang C Metformin inhibits nuclear receptor TR4-mediated hepatic stearoyl-coenzyme a desaturase 1 gene expression with altered insulin sensitivity Diabetes 2011 60 5 1493 1503 10.2337/db10-0393 21478464 PMC3292323

[B15] Xie S Lee YF Kim E Chen LM Ni J Fang LY Liu S Lin SJ Abe J Berk B TR4 nuclear receptor functions as a fatty acid sensor to modulate CD36 expression and foam cell formation Proc Natl Acad Sci U S A 2009 106 32 13353 13358 10.1073/pnas.0905724106 19666541 PMC2726407

[B16] Collins LL Lee YF Heinlein CA Liu NC Chen YT Shyr CR Meshul CK Uno H Platt KA Chang C Growth retardation and abnormal maternal behavior in mice lacking testicular orphan nuclear receptor 4 Proc Natl Acad Sci U S A 2004 101 42 15058 15063 10.1073/pnas.0405700101 15477591 PMC524065

[B17] Zhang X Schwarz EM Young DA Puzas JE Rosier RN O'Keefe RJ Cyclooxygenase-2 regulates mesenchymal cell differentiation into the osteoblast lineage and is critically involved in bone repair J Clin Invest 2002 109 11 1405 1415 12045254 10.1172/JCI15681 PMC151001

[B18] Roy ME Nishimoto SK Rho JY Bhattacharya SK Lin JS Pharr GM Correlations between osteocalcin content, degree of mineralization, and mechanical properties of C. carpio rib bone J Biomed Mater Res 2001 54 4) 547 553 11426600 10.1002/1097-4636(20010315)54:4<547::aid-jbm110>3.0.co;2-2

[B19] Lee NK Sowa H Hinoi E Ferron M Ahn JD Confavreux C Dacquin R Mee PJ McKee MD Jung DY Endocrine regulation of energy metabolism by the skeleton Cell 2007 130 3 456 469 10.1016/j.cell.2007.05.047 17693256 PMC2013746

[B20] Gutierrez S Liu J Javed A Montecino M Stein GS Lian JB Stein JL The vitamin D response element in the distal osteocalcin promoter contributes to chromatin organization of the proximal regulatory domain J Biol Chem 2004 279 42 43581 43588 10.1074/jbc.M408335200 15299011

[B21] Sierra J Villagra A Paredes R Cruzat F Gutierrez S Javed A Arriagada G Olate J Imschenetzky M Van Wijnen AJ Regulation of the bone-specific osteocalcin gene by p300 requires Runx2/Cbfa1 and the vitamin D3 receptor but not p300 intrinsic histone acetyltransferase activity Mol Cell Biol 2003 23 9 3339 3351 10.1128/MCB.23.9.3339-3351.2003 12697832 PMC153185

[B22] Javed A Gutierrez S Montecino M van Wijnen AJ Stein JL Stein GS Lian JB Multiple Cbfa/AML sites in the rat osteocalcin promoter are required for basal and vitamin D-responsive transcription and contribute to chromatin organization Mol Cell Biol 1999 19 11 7491 7500 10523637 10.1128/mcb.19.11.7491 PMC84749

[B23] Shyr CR Collins LL Mu XM Platt KA Chang C Spermatogenesis and testis development are normal in mice lacking testicular orphan nuclear receptor 2 Mol Cell Biol 2002 22 13 4661 4666 10.1128/MCB.22.13.4661-4666.2002 12052874 PMC133912

[B24] Kawai M Rosen CJ PPARgamma: a circadian transcription factor in adipogenesis and osteogenesis Nat Rev Endocrinol 2010 6 11 629 636 10.1038/nrendo.2010.155 20820194 PMC3132113

